# Diversity, Distribution, and Development of Hyperparasitic Microsporidia in Gregarines within One Super-Host

**DOI:** 10.3390/microorganisms11010152

**Published:** 2023-01-06

**Authors:** Ekaterina V. Frolova, Gita G. Paskerova, Alexey V. Smirnov, Elena S. Nassonova

**Affiliations:** 1 Laboratory of Cytology of Unicellular Organisms, Institute of Cytology of Russian Academy of Sciences, Tikhoretsky Ave. 4, 194064 St. Petersburg, Russia; 2 Department of Invertebrate Zoology, Faculty of Biology, St Petersburg University, Universitetskaya Emb. 7/9, 199034 St. Petersburg, Russia

**Keywords:** microsporidia, Metchnikovellida, hyperparasites, co-occurring infections, mixed infections, host–parasite relationships

## Abstract

Metchnikovellids (Microsporidia: Metchnikovellida) are poorly studied hyperparasitic microsporidia that live in gregarines inhabiting the intestines of marine invertebrates, mostly polychaetes. Our recent studies showed that diversity of metchnikovellids might be significantly higher than previously thought, even within a single host. Four species of metchnikovellids were found in the gregarines inhabiting the gut of the polychaete *Pygospio elegans* from littoral populations of the White and Barents Seas: the eugregarine *Polyrhabdina pygospionis* is the host for *Metchnikovella incurvata* and *M. spiralis*, while the archigregarine *Selenidium pygospionis* is the host for *M. dogieli* and *M. dobrovolskiji*. The most common species in the White Sea is *M. incurvata*, while *M. dobrovolskiji* prevails in the Barents Sea. Gregarines within a single worm could be infected with different metchnikovellid species. However, co-infection of one and the same gregarine with several species of metchnikovellids has never been observed. The difference in prevalence and intensity of metchnikovellid invasion apparently depends on the features of the life cycle and on the development strategies of individual species.

## 1. What Are Metchnikovellids?

Metchnikovellids are highly specialized microsporidia. The latter are unicellular eukaryotic spore-forming parasites of animals and some protists. Microsporidia belong to the holomycotan branch of opisthokonts [1,2]. They have a complex life cycle resulting in formation of spores with a highly elaborated invasion apparatus [3]. It is a synapomorphy of all representatives of the taxon. In typical microsporidia, the invasion apparatus consists of a set of highly specialized organelles: a polar sac-anchoring disk complex, polaroplast, coiled polar filament, and posterior vacuole [4]. The invasion apparatus of metchnikovellid spores lacks some of these organelles. In particular, instead of a long coiled polar filament, their spores possess a short, thick “manubrium” and lack a posterior vacuole. No developed polaroplast has been shown in metchnikovellid spores. Instead, they possess a tubulovesicular network at the posterior end of the manubrium [5,6,7]. Based on these characters, metchnikovellids were considered to be primitive microsporidia [8,9]. Later, this suggestion was approved by molecular studies. Recent phylogenetic and phylogenomic reconstructions placed metchnikovellids as a basal branch to the clade, embracing typical microsporidia [10,11,12].

Metchnikovellids have two types of sporogony in their life cycle: “sac-bound sporogony” and “free sporogony” [6,7]. As a result of sac-bound sporogony, a limited number of spores was formed endogenously within thick-walled spore sacs (‘cysts’ *sensu* Caullery and Mesnil [13,14]). The number of spores formed inside the sac is usually species-specific. The form and size of spore sacs is the main trait in the classification of metchnikovellids. Together with host specificity, it has been used for species distinction since the beginning of the 20th century. During free sporogony, spores are produced in the host cytoplasm (sometimes within a vacuole), without formation of spore sacs [6,9]. Free spores may differ in size and shape from sac-bound spores. Usually, the free sporogony starts before the sac-bound one, but during further development of the parasite they occur in parallel, and a heavily infected gregarine cell usually contains both free and sac-bound spores.

**Figure 1:**
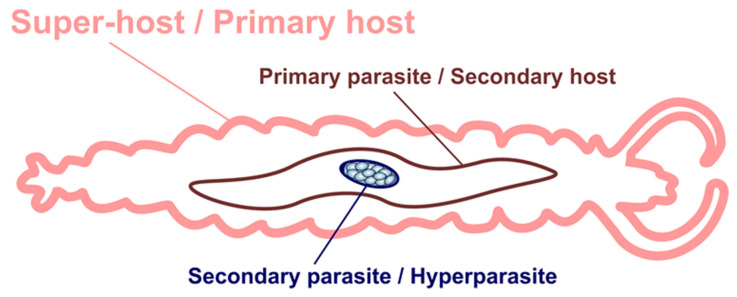
Schematic representation of a hyperparasitic system with the involvement of metchnikovellids. Polychaetes play the role of primary hosts or super-hosts. Gregarines act as primary parasites for polychaetes and secondary hosts for metchnikovellids at the same time. Metchnikovellids take the role of secondary parasites or hyperparasites.

Metchnikovellids infect gregarines that inhabit the intestines of various marine annelids. The vast majority of species are known from gregarines living in polychaetes, and a few species are known from those inhabiting sipunculids and echiurids. Hence, they are hyperparasites (or secondary parasites), i.e., the organisms that use other parasites as hosts for nourishment [15,16]. In complex parasitic systems with involvement of metchnikovellids, gregarines play the role of primary parasites (and the secondary host at the same time), while the annelid worm is referred to as the super-host or the primary host [17] (Figure 1).

Microsporidia are advanced intracellular parasites. Their incredible plasticity facilitates host switches and results in the expansion of the host range (e.g., by infection of parasites of the original host). Not surprisingly, hyperparasitism is a widespread phenomenon among this group, and hyperparasitic species are widely dispersed in the microsporidian tree. Microsporidia are known to parasitize platyhelminthes [18], in particular, trematodes [19,20,21]. Microsporidiosis has been reported in monogenean *Pseudodiplodorchis americanus* [22] and cestodes, e.g., infections caused by *Nosema helminthorum* in sheep tapeworms [23,24]. *Microsporidium acanthocephali* and *M. propinqui* are hyperparasitic microsporidia found in several species of acanthocephalans [25]. Myxosporidia are also known to be parasitized by microsporidia, either specifically [26] or facultatively [27]. Microsporidia were also found in paramyxids that parasitize marine crustaceans [28]. However, Metchnikovellida is the only group of microsporidia consisting exclusively of hyperparasites.

**Figure 2:**
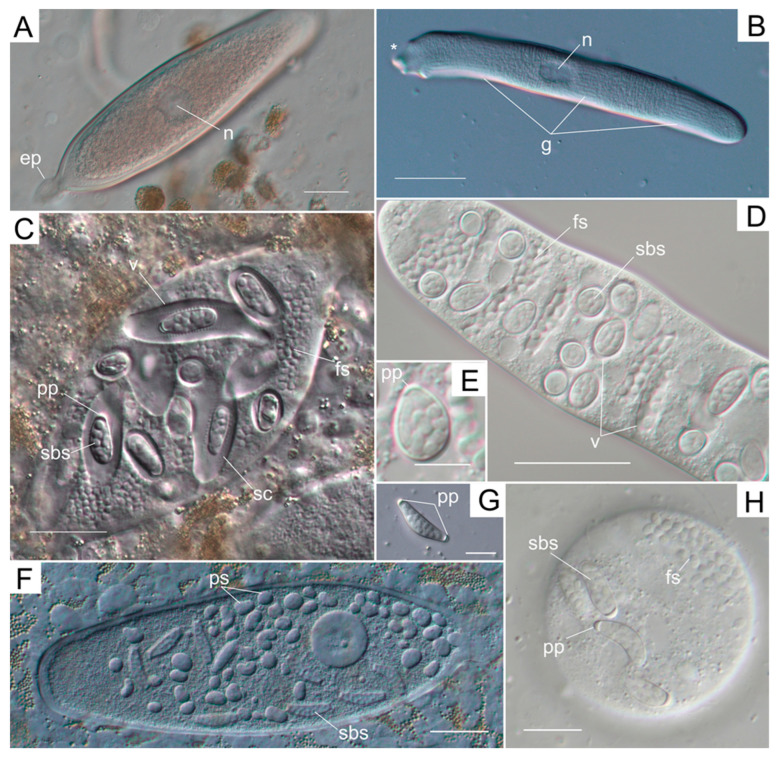
Primary and secondary parasites of the polychaete *Pygospio elegans* under Leica DM 2500 microscope equipped with DIC optics and Plan-Apo objective lenses and photographed using DFC295 digital camera (**A**–**C**,**F**,**G**) or a Nikon DS-Fi3 digital camera (**D**,**E**,**H**). (**A**) Live uninfected *Polyrhabdina pygospionis* trophozoite with epimerite (**ep**) and one nucleus (**n**); (**B**) Live uninfected *Selenidium pygospionis* trophozoite with one **n** and pellicle longitudinal grooves (**g**) seen in focal plane, asterisk marks the anterior end of the cell; (**C**) *P. pygospionis* infected with *Metchnikovella spiralis*, the hyperparasite forms the clusters of free spores (**fs**) and sac-bound spores (**sbs**), spore sacs have one polar plug (**pp**) and exterior spiral cord (**sc**), spore sacs are enclosed in vacuoles (**v**); (**D**) *S. pygospionis* filled with *M. dobrovolskiji* **fs** and **sbs**, both spore sacs and **fs** are enclosed in **v**; (**E**) spore sack of *M. dobrovolskiji* has one **pp**; (**F**) *P. pygospionis* with **sbs** and proliferative stages (**ps**) of *M. incurvata*; (**G**) isolated spore sac of *M. incurvata* with two **pp**; (**H**) *S. pygospionis* filled with *M. dogieli* **fs** and **sbs**, spore sacs with one **pp**. Scale bars: (**A**–**D**)—20 µm, (**E**)—5 µm, (**F**)—30 µm, **(G**,**H**)—10 µm.

About 30 species of metchnikovellids have been described during more than a hundred years of study. Many of them are known only from old descriptions and illustrations. The hyperparasitic lifestyle and complex population dynamics seriously complicate research of these organisms. No metchnikovellid species has ever been recorded from two or more species of gregarines and super-hosts. Therefore, they are considered to be highly specialized hyperparasites. However, it was discovered that one gregarine species can host at least two metchnikovellid species [29]. The most striking example is the parasitic system consisting of the spionid polychaete *Pygospio elegans*, a host for the eugregarine *Polyrhabdina pygospionis* (Figure 2A) and the archigregarine *Selenidium pygospionis* (Figure 2B) and their metchnikovellid parasites. *Polyrhabdina pygospionis* is a host for *Metchnikovella spiralis* (Figure 2C) and *M. incurvata* (Figure 2F,G), while *Selenidium pygospionis* can harbor *M. dobrovolskiji* (Figure 2D,E) and *M. dogieli* (Figure 2H). We monitored this system in the White Sea for over a decade [11,12,29,30,31,32,33,34]. In recent years, screenings were also initiated in the Barents Sea. The present review provides a brief summary of these studies.

## 2. Four Hyperparasites for One Super-Host: Metchnikovellids Inhabiting Gregarines from the Gut of the Polychaete *Pygospio elegans*

*Pygospio elegans* in the studied locations harbors two hosts of metchnikovellids—the eugregarine *Polyrhabdina pygospionis* and the archigregarine *Selenidium pygospionis.* In total, we have found four metchnikovellid species in these gregarines. These species differ in morphology of spore sacs and in some developmental traits, which are summarized in Table 1.

The eugregarine *P. pygospionis* hosts two metchnikovellid species. The first one is *Metchnikovella spiralis.* This species possesses oval-shaped spore sacs with one polar plug (Figure 2C). It has a unique complex structure of the spore sac, which is wrapped in a spiral cord. The latter looks like regularly arranged striations on the surface of the sac under the light microscope. The size of spore sacs is 10.3–16.5 µm in length and 5.4–7.1 µm in width [29,33]. This species has 8 oval spores per sac. Free spores are rounded or oval and are slightly smaller than sac-bound ones (Table 1). Both clusters of free spores and spore sacs are enclosed in vacuoles of unknown origin, traditionally termed “parasitophorous vacuoles”. Each spore sac is encased in an individual vacuole of a remarkably large volume. The space between the sac wall and vacuolar membrane is filled with fine filamentous material, which is probably a derivate of the external layer of the sac wall [29].

Another parasite of *P. pygospionis*, *Metchnikovella incurvata*, has bent and oblong (boomerang-shaped) spore sacs with two polar plugs (Figure 2G). These spore sacs are 22–27 µm long and 4–5 µm wide [30]. Up to 16 oval spores can be found in each spore sac. Free spores of *M. incurvata* are also oval and slightly smaller than sac-bound ones (Table 1). Both free and sac-bound sporogonies occur in direct contact with the host cytoplasm; no parasitophorous vacuoles were found (Figure 2F).

The archigregarine *Selenidium pygospionis* is the host for two other species of metchnikovellids. Of them, *Metchnikovella dobrovolskiji* has oval, irregularly oval, or pear-shaped spore sacs, with rounded ends and a thin polar plug at one end (Figure 2D,E). The sacs are 5.6–9.2 µm long and 3.3–5 µm wide [32]. Up to 12 spores per spore sac were found. Sac-bound spores and free spores are oval, and the latter are slightly larger in length (Table 1). Both spore sacs and free spores reside in vacuoles. In this species, vacuoles surround one spore sac each, and they are significantly less voluminous than those in *M. spiralis*, while the vacuoles with free spores seem to be packed with spores less tightly (Figure 2C,D).

*Metchnikovella dogieli*, the second parasite of *S. pygospionis*, has oval, sometimes slightly bent spore sacs with one polar plug (Figure 2H). Spore sacs are significantly larger than those of *M. dobrovolskiji*, measuring 9.5–34 µm in length and 4.8–9.2 µm in width [31]. The number of spores per sac varies from 7 to 18 (on average 12). Both free spores and sac-bound spores are oval; free spores are generally larger than sac-bound ones (Table 1). Free spores and spore sacs develop in direct contact with the host cytoplasm, like in *M. incurvata*.

By their morphological characters, all four studied species were classified into the genus *Metchnikovella* [8,14]. However, the definition of this genus is broad, and it unifies species which are very different in morphology of spore sacs. Our recent studies provided the first SSU rDNA sequences of named and morphologically studied organisms, nominally belonging to this genus. However, phylogenetic reconstructions showed that the genus *Metchnikovella* was genetically heterogeneous. *Metchnikovella spiralis* was robustly grouped within the clade corresponding to the family Amphiacanthidae [33], while other studied metchnikovellids formed a weakly supported clade together with *Amphiamblys* spp. [12,32,33]. Multigene phylogeny also did not provide an ultimate support for monophyly of *Metchnikovella*. We have obtained genomic data for *M. dogieli* and *M. incurvata*, but the resulting tree did not reveal them as members of a single clade [11,12]. The phylogeny of metchnikovellids needs further studies, and the relationships within the genus *Metchnikovella* will likely be seriously revised in the future.

## 3. Distribution and Prevalence of Four Metchnikovellid Species in the Host–Parasite System “*Pygospio elegans*—Gregarines”

**Figure 3:**
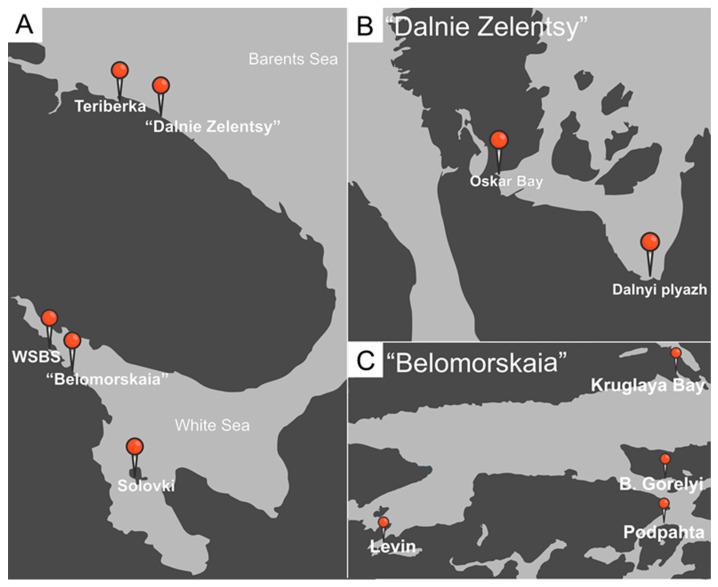
Location of sampling sites in the White and Barents Seas. (**A**) General map of sampling sites. **Teriberka** (Teriberka Bay)—69.180883, 35.190928; **Solovki** (Bolshoy Solovetsky Island)—65.020092, 35.694129; **WSBS** (White Sea Biological Station of M.V. Lomonosov Moscow State University)—66.553333, 33.104717. (**B**) Sampling sites in the Zelenetskaya Bay of the Barents Sea near the Biological Station “Dalnie Zelentsy” of the Murmansk Marine Biological Institute of Russian Academy of Sciences: **Oskar Bay**—69.120603, 36.065114; **Dalnyi plyazh**—69.111329, 36.099181. (**C**) Sampling sites in the White Sea near the Educational and Research Station “Belomorskaia” of St Petersburg University: **Kruglaya Bay**—66.338524, 33.635427; **B. Gorelyi** (Bolshoi Gorelyi Island)—66.312788, 33.629017; **Podpahta** (Podpahta strait)—66.301800, 33.629583; **Levin** (Levin reach)—66.299560, 33.465990.

In the monitored sampling sites of the Kandalaksha Gulf of the White Sea (Figure 3A,C), the prevalence of metchnikovellids (a fraction of polychaetes, containing gregarines infected by metchnikovellids) was always quite low. According to the earlier studies [29,30,34], as well to our recent observations, *Metchnikovella incurvata* was the most common species in the White Sea throughout the years (Table 2). This species might have spread from the North Atlantic with the population of its super-host—the polychaete *P. elegans* [34]. At the same time, in the Onega Gulf of the White Sea, *M. dogieli* was the most abundant metchnikovellid species in the local population of *P. elegans* screened in 2021. In the Barents Sea (connected with the White Sea by a long narrow Gorlo strait), two closely located sampling sites in the Zelenetskaya Bay (Figure 3A,B) showed the prevalence of *M. dobrovolskiji*. These data indicate a variability in the composition of metchnikovellid fauna in the polychaetes, depending on the sampling site.

Although the prevalence of metchnikovellid infection of gregarines in populations of polychaetes is usually quite low, some sites in certain periods show greater numbers of hyperparasites (Table 2). It may depend on the month of sampling or weather conditions during the season preceding the sampling. We noticed that in the unusually warm summers of 2018 and 2022, during the period of monitoring, the prevalence of metchnikovellids was lower than in the climate-wise normal years. The same site showed various prevalence of metchnikovellid species in different years, like Kruglaya Bay in the White Sea and Dalnyi plyazh in the Barents Sea (Table 2). It looks like the tripartite system “polychaetes—gregarines—metchnikovellids” depends on many variables and is highly prone to fluctuating.

A subpopulation of gregarines within a single polychaete host is called “an infrapopulation”. The prevalence of metchnikovellids differed a lot among infrapopulations: from one infected gregarine per host to dozens of infected specimens (Table 2). In some cases, almost all gregarines isolated from the gut of the worm were infected. It might depend on the amount of invasive onset obtained by the primary host, on the success of microsporidian invasion of the intact gregarines, and on the duration of microsporidian infection. The duration of maintenance of the polychaetes in the laboratory before they are inspected for parasites may also influence the results. It is important to take into account that the study of the diversity of metchnikovellids and the dynamics of hyperparasite propagation in the populations of *P. elegans* may be hampered by frequent observation of infection at the early stages when the spore sacs are not yet formed, and the hyperparasite cannot be identified by morphological means. More detailed studies of the hyperparasite prevalence in the polychaete population require the involvement of molecular methods for identification of early developmental stages of metchnikovellids (e.g., application of real-time PCR, digital droplet PCR, NGS sequencing of amplicon libraries obtained from DNA isolated from the guts of polychaete and from individually isolated gregarine cells).

## 4. Co-Occurring Metchnikovellid Infections within Gregarine Infrapopulations Inhabiting One Super-Host: Variations of Developmental Strategies

When two parasite species have the same host species, there is a chance of co-infection by these two parasites in one host organism. These parasites, in the case of mixed infection, are expected to be under resource competition, as their host represents a limited resource [35]. Mixed infections appear to be widespread among microsporidia [35,36,37,38,39,40]. In the case of co-infection of a multicellular host, interactions between microsporidia may be antagonistic: one species can moderate the effect of another one, and even exclude it from some organs, or it can negatively affect transmission of the concurrent species, or influence indirectly through the effects on the host life cycle [35,41]. Many factors seem to be important for competition in mixed microsporidian infections, such as success in host-to-host transmission, longevity of spores in the environment, and response of infected hosts to various environmental stresses, as well as competition for the same host tissue [37]. Whether mixed infections with microsporidia occur within a unicellular primary host, and what factors may be involved in the control of interspecific competition between hyperparasites, are the questions that remain to be resolved.

Two species parasitizing the same host cell have never been reported for microsporidia, and we have never seen a mixed infection of a single gregarine cell with two species of metchnikovellids. This is either a very rare event or, most probably, an impossible one. Inability of dual infection of the gregarine can be explained by a change in the structure of its pellicle after metchnikovellid invasion that would prevent the entry of other hyperparasites, or by quick depletion of host cell resources during rapid proliferation at the initial stages of microsporidian development, which also leads to the blockage of secondary infection.

In the studied complex host–parasite system, we observed mixed infections at the level of infrapopulation of gregarines persisting in one worm. The following examples of such co-occurring infections were seen: (a) infrapopulation of *P. pygospionis,* infected either with *M. incurvata* or with *M. spiralis* [29,33], (b) infrapopulation of *S. pygospionis*, infected either with *M. dogieli* or with *M. dobrovolskiji* [32]. Co-occurring metchnikovellid infections were also common for eugregarine and archigregarine infrapopulations from the same polychaete host. When the host was parasitized with both *P. pygospionis* and *S. pygospionis,* we observed the cases when the first species was infected with *M. incurvata,* while the second one contained *M. dogieli*. We also observed the cases of co-occurring infections caused by *M. spiralis* and *M. dogieli* within one specimen of the super-host. Our genomic studies showed that up to three metchnikovellid species can be detected in the samples from one specimen of the primary host [42].

Apparently, mixed infections within a primary host infrapopulation are common for metchnikovellids. Sequences of the SSU rDNA gene of two distinct species of metchnikovellids were amplified from infected archigregarines sampled from one specimen of the polychaete *Travisia forbesii*. Two metchnikovellid sequences were also detected in the infected gregarines *Ancora sagittata* from a specimen of the polychaete *Capitella capitata* [43].

Each gregarine species of *P. elegans* can host two metchnikovellid species that demonstrate two different developmental patterns. The major difference is the presence or absence of parasitophorous vacuoles, surrounding spore sacs or groups of free spores. The role and origin of these vacuoles is not yet understood. It can be hypothesized that the presence of the vacuole can physically limit the number of spore sacs and free spores produced within a gregarine cell. They probably affect the limitation of spore sac number per gregarine host in the case of infection caused by *M. spiralis*, where a major part of the host cell cytoplasm is occupied by parasite-containing vacuoles [33]. On the other hand, *M. dobrovolskiji* has much smaller spore sacs, which occupy a smaller volume of the host cytoplasm. However, in absolute numbers, this species produces the highest number of spore sacs per host cell (Table 1). The small size of the sacs of this species can apparently contribute to the accumulation of more infectious onset within a single gregarine cell. *Metchnikovella incurvata* and *M. dogieli* tend to fill the entire host cell with spore sacs and free spores. This results in the deformation of the gregarine, followed by the rupture of its pellicle (this may happen even before maturation of spores and spore sacs). Such a massive and destructive production of spores and spore sacs has never been observed for the species retaining the vacuoles. This indicates that different metchnikovellid species exploit different development strategies. *Metchnikovella spiralis* and *M. dobrovolskiji* produce a smaller number of infectious onsets, which are retained in the vacuoles. These hyperparasites maintain the integrity of the host cell until complete maturation of spores and spore sacs before their exit into the environment. In this way, they seem to be able to achieve success without spending additional resources on production of a larger number of spore sacs and spores. It is interesting that metchnikovellids with both types of development strategies inhabit both studied primary hosts, the eugregarine *P. pygospionis* and the archigregarine *S. pygospionis* (Table 1).

## 5. Impact of the Metchnikovellids on Gregarines

At the early stages of infection with metchnikovellids, gregarines maintain a typical cell shape and mobility. At the later stages of metchnikovellid development, when the host cell is filled with spore sacs and free spores, gregarine cells are getting deformed. The cells of *P. pygospionis* tightly packed with the hyperparasites become wider, while retaining their ability to glide [30]. The spore sacs and free spores of *M. incurvata* fill the gregarine cell so densely that almost no host cytoplasm and amylopectin granules remain visible under a light microscope. In some gregarines, very tightly packed with hyperparasites, even the nucleus is not visible. The other parasite of this eugregarine, *M. spiralis*, does not seem to be able to produce so many spore sacs. Nevertheless, the gregarines infected with *M. spiralis* become significantly wider, as spore sacs are enclosed in voluminous vacuoles that occupy a large volume of the host cell.

In the case of archigregarines parasitized with *M. dogieli*, the difference between infected and uninfected cells is even more obvious. Uninfected cells of *S. pygospionis* are elongated, vermiform, and slightly flattened, with a pointed anterior end and rounded posterior end. The entire surface of archigregarines bears a number of longitudinal grooves (Figure 2B). These gregarines bend their bodies smoothly, almost like nematodes. They are constantly in motion, being either attached to the tissues of the host or free. In archigregarines infected with *M. dogieli*, the body becomes strongly shortened, thickened, and uneven, and cortical grooves are not defined. Their motility is clearly restricted, and the cell is not able to curve the body to the full extent [31]. *S. pygospionis* infected with *M. dobrovolskiji* usually tend to maintain the form and motility, even if there are many spore sacs and free spores in the host cytoplasm. This is probably due to the small size of the spore sacs of this species [32].

**Figure 4:**
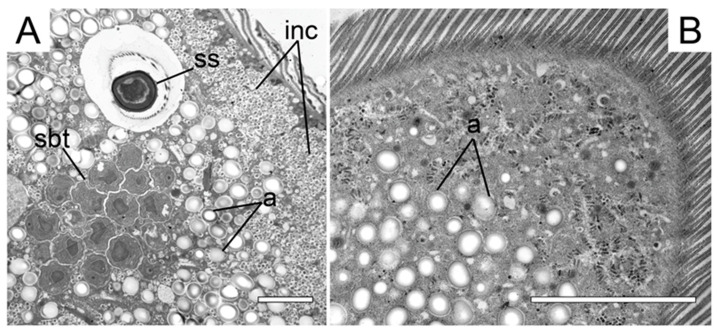
Fine structure of the cytoplasm in infected and healthy *Polyrhabdina pygospionis*. (**A**) Eugregarine infected with *Metchnikovella spiralis*. Spore sac (**ss**) and sporoblasts (**sbt**) are seen in the cytoplasm along with some amylopectin granules (**a**), while the periphery of the cell is full of small round inclusions (**inc**). (**B**) Healthy eugregarine. There are numerous amylopectin granules in the cytoplasm and no small inclusions in the periphery. Scale bars: (**A**)—3 µm, (**B**)—5 µm.

Analyses of the two metchnikovellid genomes showed that metabolic capabilities of metchnikovellids are as reduced as in higher microsporidia, which suggests their dependence on gregarine host metabolites [10,11]. Electron-microscopic studies show that in infected gregarine cells, the vesicles of the endoplasmic reticulum aggregate around the parasites [30,44]. The gregarine nuclei were never seen invaded, but some researchers mentioned that the parasite might induce formation of secondary nucleoli [6]. In infected *P. pygospionis* cell, infection with metchnikovellids caused formation of numerous small, rounded inclusions at the periphery of the cell (Figure 4A). Non-infected gregarines never contained such inclusions (Figure 4B). Therefore, metchnikovellids seem to affect the gregarine cell and re-direct the host metabolism to serve the needs of their proliferation and sporogony. It is likely that the remaining resources are insufficient for subsequent development and gametogenesis of the gregarine itself.

## 6. The Impact of Metchnikovellids on the Host–Parasite System “*Pygospio elegans*—Gregarines” with Notes on Hypothetical Life Cycle of Metchnikovellids and Presumable Ways of Transmission

Hyperparasites are believed to play a role in controlling the quantity and evolution of their secondary hosts, as primary parasites do for the primary hosts [45]. This means that hyperparasites can help to reduce the pathogenic impact of the primary host on the super-host population by controlling its number [15].

It has been shown that gregarines influence their hosts in numerous ways [46]. Little is known about the impact of gregarines on their aquatic, especially marine, hosts, but studies on the relationships between gregarines and terrestrial insects are numerous. Most gregarine infestations were considered benign, though for some species their negative effect on the host development, fitness, and longevity has been shown [47,48,49]. Some gregarines exhibited positive effects on their hosts [50,51,52]. The essential role of gregarine infection in growth of the host larvae, longevity, and chance of inbreeding was demonstrated [46]. Some gregarines have been suggested to be essential for fitness of their hosts [53]. Many studies also demonstrated no effect at all [54]. Our studies [55,56], and the observations performed by Hiillos with co-authors [57], showed that the gregarines were widely distributed in *P. elegans* populations, in which most polychaete specimens harbored these parasites. The prevalence of gregarines *S. pygospionis* and *P. pygospionis* varied among populations of polychaetes, which is a common feature of apicomplexan parasites [57]. Infection with metchnikovellids is likely lethal to gregarines and therefore should efficiently regulate the size of gregarine infrapopulations.

There is no information on the gregarine life stages infected with metchnikovellids other than trophozoites, suggesting that infected trophozoites may lose the ability to form cell-to-cell contact in the syzygy stage or fail to continue development even if the syzygy is formed. In addition, metchnikovellids hardly leave enough resources for the gregarines to reproduce, as was mentioned above.

**Figure 5:**
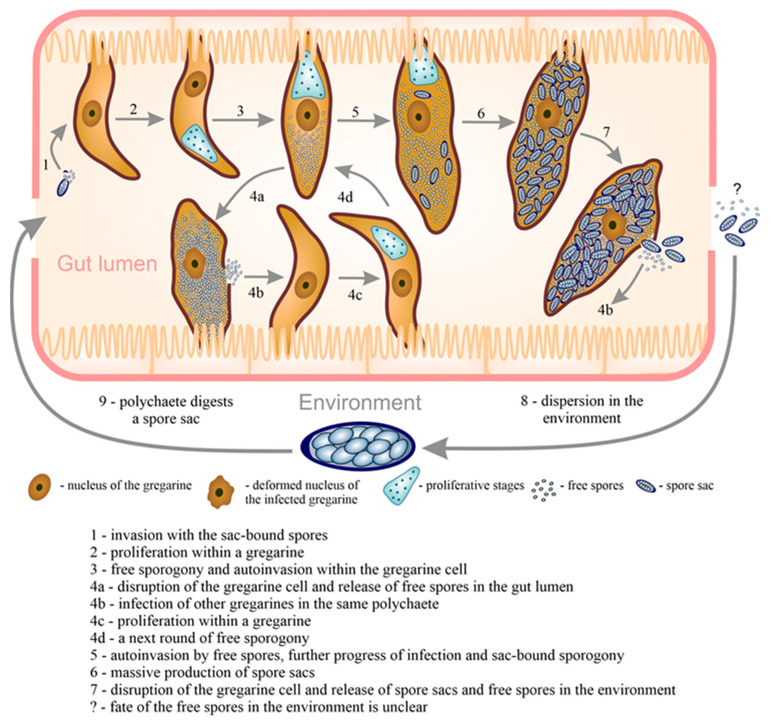
Life cycle of a metchnikovellid and its dispersion within a polychaete host and in the environment. The scheme reflects our current working hypothesis about the development of hyperparasite and its transmission. After invasion (**1**), rapid proliferation of hyperparasites begins in the cytoplasm of gregarine cell (**2**), followed by free sporogony (**3**). Free spores disseminate within the infected gregarine that results in massive production of spores (accumulation of invasive onset) and destruction of the gregarine. As a consequence, free spores disperse in the gut and infect other gregarines (**4**). Subsequent infections lead to the production of both free spores and spore sacs (**5**,**6**). Spore sacs disperse in the polychaete gut lumen after disruption of the gregarine (**7**). Spore sacs disperse into the environment through the intestine of the polychaetes (**8**). The cycle is initiated again after a spore sac is ingested by the polychaete (**9**).

The following scheme of metchnikovellid infection can be suggested: a primary host (a polychaete) consumes a spore sac. The plug of the sac opens in the intestine, then the spores released from the sac infect the primary parasites (the gregarine trophozoites) in the gut lumen (Figure 5). The role of free spores in the primary infection is not clear. It is not established if they can survive in the environment for a time, sufficient to be consumed by a super-host (annelid worm).

The mechanism of metchnikovellid invasion via spores is not yet known. In microsporidia with the classic invasion apparatus, the polar tube everts after discharge, interacts with the plasma membrane of the host cell, and causes its invagination, the “invasion synapse”, at the site of the contact. In this invagination, the polar tube can either penetrate the plasma membrane or interact with it. In the first case, penetration of the plasma membrane results in its local disruption and injection of the sporoplasm (an invasive onset of microsporidia), so it occurs in direct contact with the host cytoplasm. In the second case, the sporoplasm itself interacts with the host plasma membrane, forcing it to initiate phagocytosis. It leads to the formation of a parasitophorous vacuole derived from the host plasma membrane, which surrounds the sporoplasm [58]. There are two hypotheses on the ways of penetration of the metchnikovellid sporoplasm into the gregarine cell. The direct injection of short and wide manubrium through thick gregarine pellicle can hardly happen mechanically [29]. It can be proposed that manubrium adheres to the pellicle and excretes enzymes (that could be stored in the vesicles in the lamellar fold at the posterior end of manubrium). These enzymes disintegrate the pellicle and then the sporoplasm is injected into the host cell cytoplasm. According to an alternative idea, the sporoplasm causes invagination of the host plasma membrane in the contact zone between the manubrium and the area of the gregarine micropore characterized by thinned pellicle [30,34]. It should be noted that archigregarines *S. pygospionis* and eugregarines *P. pygospionis* have numerous micropores on their cell surface, at the bottom of cortical grooves and on the sides of epicytic folds, respectively [55,56]. A similar mechanism of invagination of the host plasma membrane in the contact zone between the penetration tube and the host cell (a kind of invasion synapse) is suggested for aphelids, holomycotan parasitoids of algae, closely related to fungi, and *Rozella* spp., parasitoids of zoosporic fungi and oomycetes, closely related to microsporidia [59].

After invasion, rapid proliferation of parasites starts in the cytoplasm of gregarine cell, followed by sporogony. The free sporogony usually precedes the sac-bound one. Free spores are most probably responsible for auto-invasion of the infected gregarine at the early stages. When the gregarine filled with spores breaks, free spores disperse in the gut and may infect other gregarines. This is the hypothetical mechanism of the primary dispersion within the infrapopulation of gregarines. Subsequent infections lead to production of free spores and spore sacs for accumulation of invasive onset. It is possible that the type and rate of sporogony vary according to the capacity of the gregarine host. Spore sacs disperse into the environment through the intestine of the primary host after gregarine death, while free spores can continue spreading infection within the infrapopulation. Certainly, a fraction of free spores also appears in the environment, but their further fate is not clear. Thus, the general strategy for metchnikovellid infection is a passive horizontal dispersion, involving both the environment and the primary host. Infection requires the retention of the invasive onset, the spore sacs, in the biotope. The primary host (a polychaete) must have a secondary host (the gregarines) in its intestine to carry out the metchnikovellid life cycle.

Besides the apparent complexity of this parasitic system, another characteristic of metchnikovellids is high diversity. In about ten years of studies and observations made in several locations, we reisolated one and discovered three new species and showed that one gregarine species can be a host for at least two metchnikovellid species. In fact, we mentioned several more species of metchnikovellids from other hosts in these habitats, which have not yet described systematically. It is even hard to predict how many more new species might be found in the case of targeted, wider-scale studies. Evidently, the number of metchnikovellid species is much higher than the ca 30 species currently known.

## 7. Conclusions

Metchnikovellids from the gregarines parasitizing *Pygospio elegans* are unevenly distributed in the populations of polychaetes and the infection outbreaks occur sporadically, which is usual for this group of hyperparasites. The prevalence of metchnikovellid infection is low. That is why the diversity of metchnikovellids remains heavily underexplored. The more we study the parasite fauna of gregarines from a certain primary host species, the more hyperparasite species we find. It is likely that there are a lot of yet-unknown metchnikovellid species that await discovery.

It remains unclear whether there is a limit to the number of species of metchnikovellids that can parasitize a particular gregarine species. Host range is one of the traits traditionally used for identification of metchnikovellids. However, we have shown that one super-host (e.g., polychaete) can harbor an infrapopulation of gregarines infected with several metchnikovellid species. Therefore, it is crucial to complement morphological traits with molecular and ecological data for descriptions of a new species. Proliferative stages should be identified at the molecular level to get the correct data on the prevalence of metchnikovellid infection.

Different metchnikovellid species parasitizing the same gregarine host demonstrate diverse morphological traits and developmental patterns. They can apply two alternative strategies of development: production of spore sacs and free spores either in direct contact with the gregarine cytoplasm or within the vacuole. In the first case, spore production is so intensive that the hyperparasite completely occupies the cytoplasm of the gregarine cell and finally causes a rupture of the gregarine pellicle. If destruction of the host cell occurs too early, immature spores are released into the environment. In the case of species retaining parasitophorous vacuoles throughout the life cycle, the intensity of production of invasive stages is usually not so high, and integrity of the host cell is maintained until complete maturation of spores and spore sacs. Thus, the difference in prevalence and intensity of invasion by the hyperparasites seems to depend on their morphological and developmental features.

It is possible to suggest that some metchnikovellid species are more successful than others in different geographical sites and/or in different seasons of the year. Although co-occurring infections within the infrapopulation of gregarines can take place, mixed infections of the same gregarine cell with several metchnikovellid species have never been seen. Metchnikovellid infection is lethal to gregarines, so potentially these hyperparasites can control the density of gregarine population within the super-host preventing thus over-infection with gregarines in a single worm and its death. Thus, metchnikovellid infections stabilize the whole hyperparasitic system, being beneficial for all its members. It explains why such tripartite systems (polychaete—gregarine—metchnikovellid) are ubiquitous in certain marine environments.

## 8. Future Perspectives

Co-occurring (or mixed) infections of a gregarine infrapopulation with several metchnikovellid species deserve special attention in the future. How they affect the host and super-host survival, and if there is a competitive advantage for one of the metchnikovellid species, or if their interactions are synergetic, are the key questions for future studies. The answers to these questions may shed light on the diversification of metchnikovellids and co-evolution of gregarines and their hyperparasitic microsporidia.

Targeted studies that combine observational methods with new methods, such as eDNA techniques, are necessary to deal with the early stages of infection. Sampling should become more intensive to get a comprehensive set of data for proper statistical analysis. Greater attention should also be paid to the ratio of metchnikovellid species in mixed infections in gregarine infrapopulations.

The frequent occurrence of the infections caused by different metchnikovellid species in gregarines within one super-host and identification of several new hyperparasite species from one super-host suggest that traditional descriptions of metchnikovellid species based on the host range cannot be considered valid without modern morphological and molecular studies. Studies of metchnikovellids nowadays require the use of high-quality light microscopy, electron microscopy and single-cell manipulations with individually isolated cells of infected gregarines, followed by the application of methods of single-cell genomics.

## Figures and Tables

**Table 1:** Morphological diversity and GenBank references of the metchnikovellids from *Pygospio elegans*.

Hyperparasite	*Metchnikovella* *incurvata*	*Metchnikovella* *spiralis*	*Metchnikovella* *dogieli*	*Metchnikovella* *dobrovolskiji*
Secondary host	*Polyrhabdina pygospionis*	*Polyrhabdina pygospionis*	*Selenidium pygospionis*	*Selenidium pygospionis*
Size ^1^ of spore sacs, μm	22–27 × 4–5	10.3–16.5 × 5.4–7.1	9.5–34 × 4.8–9.2	5.6–9.2 × 3.3–5
Form of spore sac	boomerang-shaped	oval	oval, sometimes bent	oval or pear-shaped
Number of polar plugs	two	one	one	one
Number of spore sacs per host cell	about 30 in one focal plate	20	up to 24	up to 41
Sac-bound spores (number per sac; morphology; size ^1^, μm)	up to 16; oval or ovoid; 3.6 × 1.8	8; oval; 2.4–3.5 × 2.4–2.9	7–18 (often 12–14); oval; 2.2–3.0 × 1.4–2.9	up to 12; oval; 1.3–2.4 × 0.9–1.6
Free spores (morphology; size ^1^, μm)	oval or ovoid; 3.7 × 1.8	rounded or oval, slightly angular at the top of the polar cap; 2.5–3.5 × 2.1–2.3	oval or ovoid, sometimes with a small bulge on one side; 2.2–3.3 × 1.3–3.7	oval; 1.2–3.1 × 1.1–1.7
Spore sac enclosed in the individual vacuoles	no	yes	no	yes
Free spores enclosed in the vacuoles	no	yes	no	yes
GenBank	OK155996	MW344837	OK155994	OP225322
References	[11,30]	[29,33]	[12,31]	[32]

^1^ Measurements for all species are provided for live spores and spore sacs.

**Table 2:** Occurrence and prevalence of metchnikovellids and their gregarine hosts in the polychaetes *Pygospio elegans* collected from the White and Barents Seas.

Site	Year	N	with P	with S	Mix. P + S	Mi	Ms	St. P	Md	Mj	St. S	Mi + Ms	Mi + Md	Ms + Md	Ms + Mj	Mj+ Ms+Mi
White Sea																
Levin	2018	220	112	67	44	0	0	0	0	0	0					
	2019	34	31	16	15	0	0	0	0	0	0					
B. Gorelyi	2018	18	15	16	15	0	0	0	0	0	1					
	2019	85	76	59	44	1	0	2	1	1	0					
Kruglaya Bay	2019	115	87	90	85	3	0	6	0	4	5					1 *
	2020	26	16	7	5	1	1	0	0	0	0					
	2021	10	9	9	8	0	0	0	0	0	0					
Podpahta	2019	98	87	81	71	11	2	13	4	0	7	1	2			
Solovki	2021	16	15	14	13	0	1	3	5	2	5			1		
WSBS	2021	5	5	5	5	2	0	0	0	0	0					
Barents Sea																
Dalnyi plyazh	2020	8	8	7	7	0	1	0	2	0	1			1		
	2021	136	121	132	117	3	4	19	7	15	40				1	
	2022	66	55	51	46	0	0	0	2	1	29					
Oscar Bay	2021	71	70	55	51	2	2	3	2	13	6				1	
	2022	32	28	26	24	0	0	0	0	0	5					
Teriberka	2021	17	15	4	4	0	0	0	0	0	0					

All data (except *) were obtained from light-microscopical observations; in the case of (*) the data were obtained from genomic survey. **Abbreviations** (horizontally in the table header): **N**—a total number of worms analyzed; (**with P**)—a number of polychaetes infected with *Polyrhabdina pygospionis*; (**with S**)—with *Selenidium pygospionis*; (**Mix. P + S**)—with both gregarine species—*P. pygospionis* and *S. pygospionis*; (**Mi**)—a number of gregarines *P. pygospionis* infected with *Metchnikovella incurvata*; (**Ms**)—with *M. spiralis*; (**St. P**)—a number of gregarines *P. pygospionis* with unidentified metchnikovellid infection, hyperparasitic species has been left unidentified at the species level as it was found at the proliferative stage; (**Md**)—a number of gregarines *S. pygospionis* infected with *M. dogieli*; (**Mj**)—with *M. dobrovolskiji*; (**St. S**)—a number of gregarines *S. pygospionis* with unidentified metchnikovellid infection; (**Mi + Ms**)—a number of polychaetes with co-occurring infections of gregarines with *M. incurvata* and *M. spiralis*; (**Mi + Md**)—with *M. incurvata* and *M. dogieli*; (**Ms + Md**)—with *M. spiralis* and *M. dogieli*; (**Ms + Mj**)—with *M. spiralis* and *M. dobrovolskiji*; (**Mj + Ms + Mi**)—with *M. dobrovolskiji*, *M. spiralis*, and *M. incurvata*.

## Data Availability

Not applicable.
